# Infiltrative retroperitoneal metastasis causing obstructive uropathy and gastric outlet obstruction secondary to an occult sigmoid colon adenocarcinoma: a rare case report and literature review

**DOI:** 10.1097/RC9.0000000000001196

**Published:** 2026-10-26

**Authors:** Abdallah Abd Ali, Ali Abd Ali, Nader Moeinvaziri, Raheleh Sharifian Paghaleh

**Affiliations:** a School of Medicine, Shiraz University of Medical Sciences, Shiraz, Iran; b Department of Surgery, Laparoscopy Research Center, Shiraz University of Medical Sciences, Shiraz, Iran

**Keywords:** case report, gastric outlet obstruction, obstructive uropathy, retroperitoneal fibrosis, sigmoid adenocarcinoma

## Abstract

**Introduction:**

Colorectal cancer most often presents with gastrointestinal problems such as obstruction and abdominal pain, or is detected early by screening colonoscopy. Retroperitoneal metastasis that mimics the findings of retroperitoneal fibrosis is rare and can cause diagnostic challenges, especially in patients in whom contrast-enhanced imaging is contraindicated.

**Case presentation:**

A 49-year-old woman was admitted with severe bilateral flank pain, oliguria, intermittent nausea, and loss of appetite without abdominal distention or constipation. Due to a known allergy to contrast media, a non-contrast CT scan was performed and showed bilateral hydronephrosis and evidence of retroperitoneal fibrosis. Although double-J ureteral stents were inserted, the patient’s renal function progressively deteriorated, requiring hemodialysis. After that, the patient experienced intractable vomiting, and the evaluation confirmed gastric outlet obstruction (GOO) caused by mass effect from a retroperitoneal mass. Magnetic resonance imaging findings continued to favor retroperitoneal fibrosis despite the presence of some suspicious pulmonary nodules. Surgical intervention with retroperitoneal biopsy was performed, during which an incidental sigmoid mass was detected and resected. Histopathology revealed well-differentiated sigmoid adenocarcinoma with retroperitoneal metastasis.

**Discussion:**

Malignancy-associated retroperitoneal fibrosis faces critical diagnostic challenges due to its radiologic findings that can mimic a benign fibroinflammatory process. Unlike previous reports that focus on retroperitoneal fibrosis in patients with known primary malignancies, this case presented without a history or symptoms of colorectal cancer. Additionally, the inability to perform a contrast-enhanced CT scan contributed to a delayed diagnosis. Failure of ureteral stenting and GOO symptoms were two main indicators of infiltrative malignant disease. Timely tissue diagnosis remains essential for atypical manifestations of retroperitoneal fibrosis.

**Conclusion:**

Occult sigmoid adenocarcinoma may rarely present with multi-level obstructive complications, even without typical large-bowel obstruction *symptoms*. Timely diagnosis and intervention require interdisciplinary collaboration to achieve critical, life-saving outcomes.

## Introduction

Colorectal cancer is considered one of the most common cancers worldwide. Most symptoms are gastrointestinal (GI), including changes in bowel habits, rectal bleeding, or iron-deficiency anemia. The cancer is usually detected early by colonoscopy^[^1–3^]^.

In rare cases, colorectal cancers may present as extraluminal manifestations due to metastatic processes. Retroperitoneal involvement is uncommon and, in severe cases, can lead to compression of adjacent structures and cause urinary and GI obstructions^[^4–7^]^.

Retroperitoneal fibrosis (RPF) can radiologically mimic malignant infiltration, especially in the absence of contrast-enhanced imaging ^[^7,8^]^.

HIGHLIGHTS

- Occult sigmoid adenocarcinoma may initially present without gastrointestinal symptoms or altered bowel habits.
- Retroperitoneal metastatic infiltration can radiologically mimic retroperitoneal fibrosis, particularly when contrast-enhanced imaging is unavailable.
- Simultaneous dialysis-dependent obstructive uropathy and gastric outlet obstruction represents an unusual presentation of colorectal cancer.
- Failure of double-J ureteral stenting should raise suspicion for malignant retroperitoneal encasement rather than benign fibrosis.

Here, we report a case of occult sigmoid adenocarcinoma in a 49-year-old woman presenting with dialysis-dependent obstructive uropathy, gastric outlet obstruction (GOO), contrast media allergy, and imaging findings suggestive of RPF, without change in bowel habit, GI bleeding, or anemia. This case highlights the diagnostic challenges in a patient presenting with atypical features of sigmoid cancer for whom contrast-enhanced imaging is contraindicated. This case report has been prepared in line with the SCARE checklist ^[^9^]^.

## Case presentation

### Patient information and clinical findings

Our patient, a 49-year-old female, came to our emergency department suffering from progressive bilateral flank pain for a few weeks, a significant decrease in urine output, intermittent nausea, and mild loss of appetite. Her surgical history is significant for a laparotomy due to an ectopic pregnancy. She was receiving irregular doses of progesterone-only contraceptive injections. She denied smoking or drug use, but reported a severe allergy to iodinated contrast media. The patient’s medical history noted that during a previous CT examination performed to investigate the aforementioned symptoms, she developed a generalized skin rash following intravenous contrast administration. The rash resolved after treatment with an antihistamine.

### Initial diagnostic assessment and intervention

The patient repeatedly presented to a local clinic for her symptoms but was managed for recurrent urinary tract infection (UTI) without significant improvement. She then decided to come to our hospital due to worsening symptoms.

Due to her contrast allergy, an abdominopelvic computed tomography (CT) scan without contrast was performed (Fig. 1). The imaging revealed severe bilateral hydronephrosis (Fig. 1B), prompting insertion of bilateral double-J ureteral stents by the urology department, given suspicion of obstructive uropathy causing acute kidney injury (AKI). However, urine output was still low, and creatinine levels continued to rise, accompanied by new-onset intractable vomiting (Table 1).

**Figure 1.**
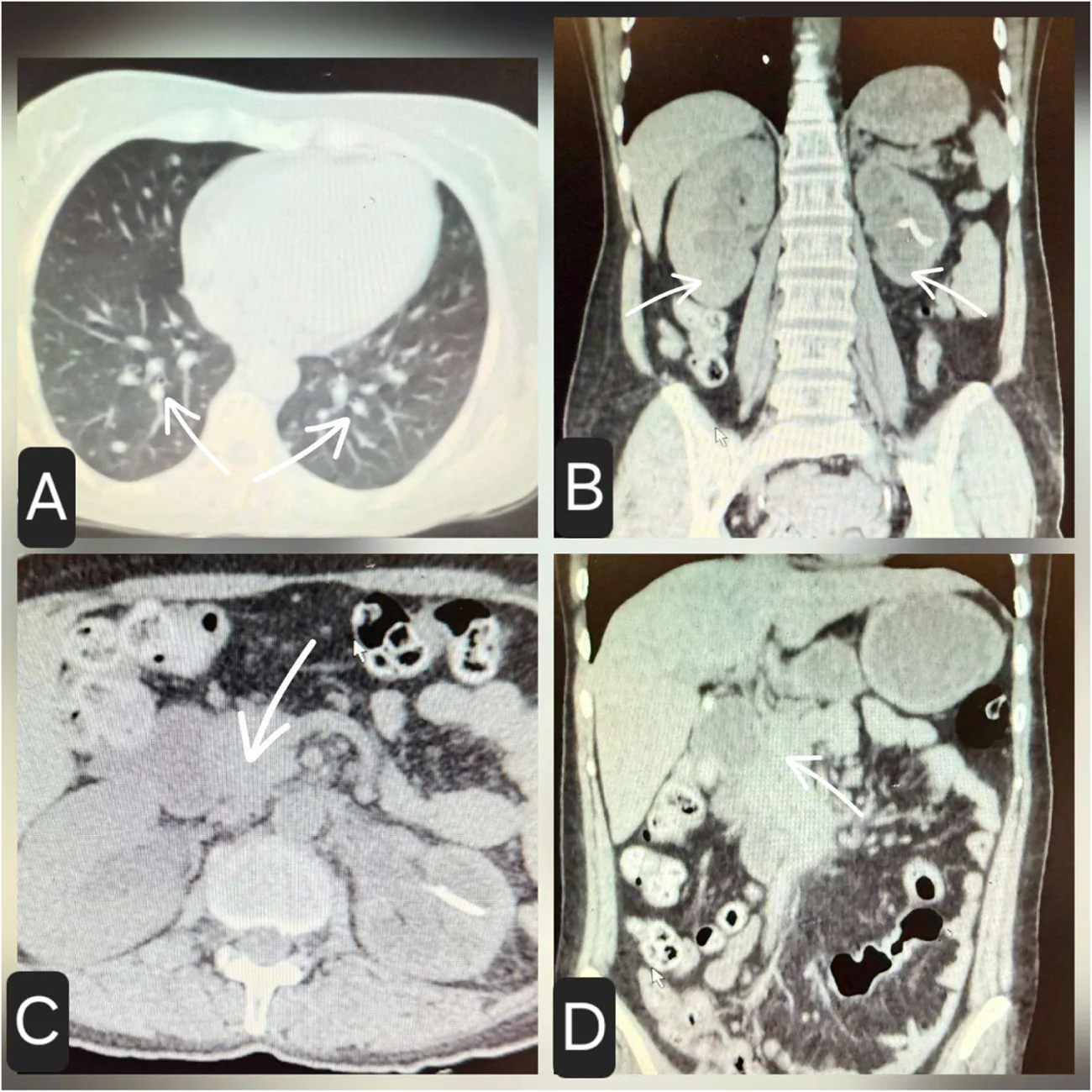
Patient’s CT scan demonstrating pulmonary nodules (A, arrows), hydronephrosis (B, arrows), and a retroperitoneal mass causing a pressure effect on the third portion of the duodenum (C and D, arrows).

**Table 1:** Serial laboratory findings throughout the clinical course.

Test	Admission	Double J insertion		Nephrostomy insertion and hemodialysis sessions		After surgery	At discharge
		After 1st day	After 2nd day	At the beginning	After multiple sessions		
BUN (mg/dL)	24	36	45	64	47	23	28
Cr (mg/dL)	2.12	5.96	8.26	6.08	1.91	1.11	1.02
PT (sec)	15.8	-	-	-	16.4	-	-
PTT (sec)	36	-	-	-	39	-	-
INR	1.19	-	-	-	1.23	-	-
WBC (white blood cells) (×10^3^)	10.7	16.3	19.2	10.3	10.8	9.5	8.8
Hb (g/dL)	10.6	10.2	8.3	7.1	7.6	9.1	8.8
Plt (×10^3^)	283	314	221	257	232	493	460

### Clinical deterioration and advanced imaging

About 3 days after bilateral double-J insertion, due to worsening AKI, the patient was transferred to the internal medicine service for further management, where her deteriorating condition required hemodialysis sessions.

Despite this, nausea and intractable vomiting continued to worsen. Reassessment of the CT scan identified a mass-like lesion in the retroperitoneal area and distortion of the D2 and D3 segments of the duodenum, which raised suspicion of GOO (Fig. 1C, D). The GOO was then confirmed by upper endoscopy and a barium swallow study. The General Surgery service was then consulted. During this time, the patient’s creatinine level was still increasing, requiring three sessions of hemodialysis weekly (Table 1). Additionally, the malpositioned and nonfunctional double-J stents required bilateral percutaneous nephrostomy placement.

For further evaluation, we performed a non-contrast CT scan and a subsequent magnetic resonance imaging (MRI) that showed a diffuse retroperitoneal infiltrative process without colonic wall thickening or a discrete primary mass. Incidentally, the CT scan revealed suspicious pulmonary nodules, which raised concern for a metastatic process of unknown origin.

The CT scan findings were consistent with complete obstruction of the third portion of the duodenum due to an infiltrative retroperitoneal process, resulting in GOO and dilatation of the first and second portions of the duodenum. These findings were further confirmed by our intraoperative observations, which showed this process encasing adjacent structures (Fig. 2).

**Figure 2.**
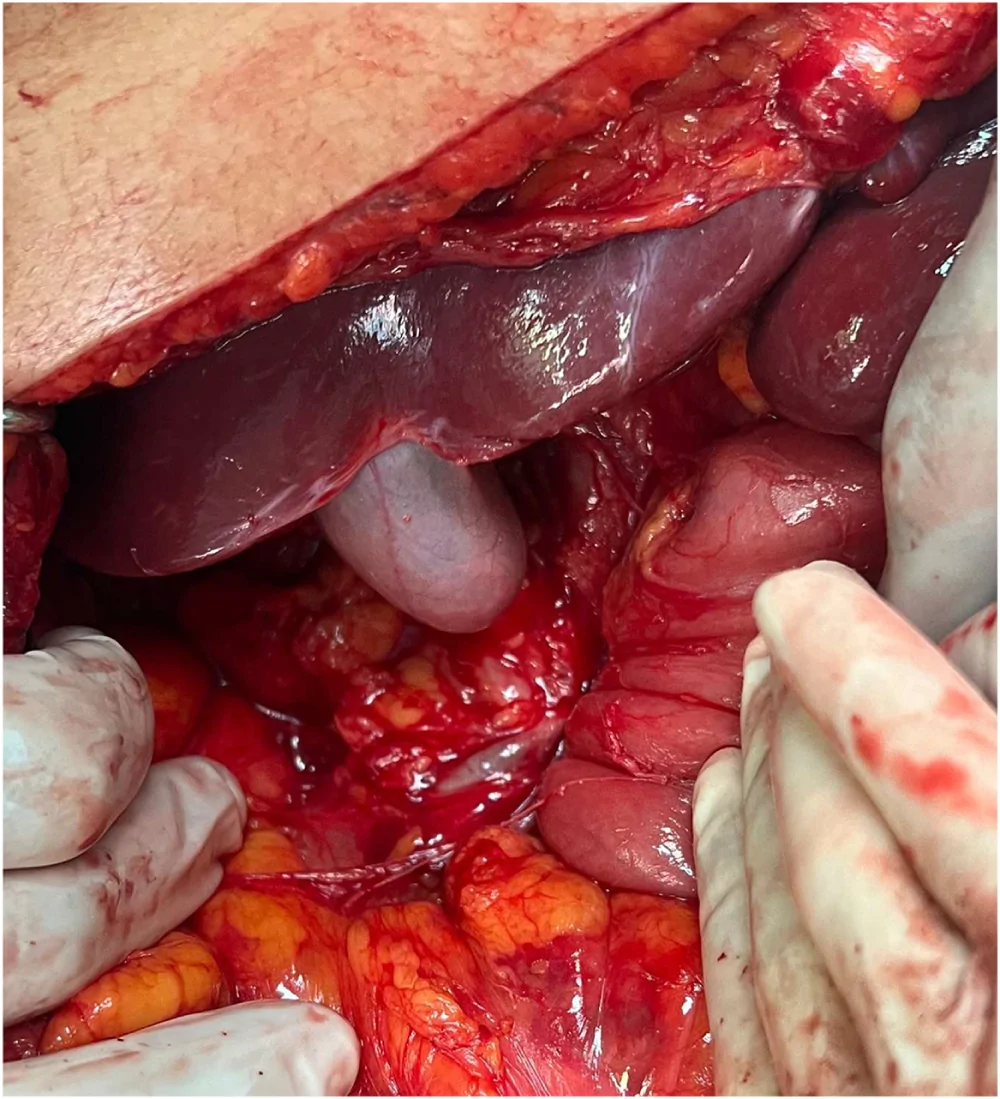
Intraoperative view showing a retroperitoneal infiltrative process encasing adjacent structures.

### Surgical intervention and pathology

Because of the previously confirmed symptomatic GOO, surgical exploration was performed, a loop gastro-jejunostomy bypass was created, and a biopsy was obtained from the retroperitoneal mass compressing the duodenum and both ureters (Fig. 2).

During exploration of different regions of the abdomen to identify any seeding or metastatic lesions, a large tumor was unexpectedly discovered in the sigmoid colon, which had nearly occluded the entire lumen.

Given the large size of the sigmoid tumor and its near-complete luminal obstruction, with a high likelihood of progression to complete obstruction in the near future, a decision was made to perform a palliative sigmoidectomy and an end colostomy (Hartmann’s procedure). As the patient remained hemodynamically stable throughout the operation, resection was considered feasible. However, considering the patient’s overall clinical condition and the lack of adequate bowel preparation, primary colorectal anastomosis was deemed inappropriate and was therefore not performed.

On the second postoperative day, oral intake was initiated with a surgical diet and gradually advanced according to the patient’s tolerance. The patient tolerated the diet well, and the colostomy became functional on the fourth postoperative day. The subsequent postoperative course was uneventful.

Histopathology confirmed a well-differentiated (G1) sigmoid adenocarcinoma measuring 3.7 × 2 × 1.6 cm, invading through the visceral peritoneum (pT4a). Lymphovascular and perineural invasion were present, with three tumor deposits. One of 10 examined lymph nodes was involved (pN1a). Proximal, distal, and circumferential/mesenteric margins were free of tumor. The retroperitoneal lesion was involved with adenocarcinoma, while the omentum was free of tumor. The reported pathological stage was pT4aN1aMx, at least stage IIIB.

## Discussion

### Overview of colorectal cancer

Colorectal cancer is one of the most common malignancies worldwide, and it usually presents with GI symptoms such as changes in stool frequency or consistency, hematochezia, or iron-deficiency anemia. It is often detected early by screening colonoscopy. However, in rare cases, colorectal cancer may present with extraluminal manifestations, especially in advanced stages of the disease or when tumors grow predominantly outward through the bowel wall rather than causing significant intraluminal narrowing or mucosal ulceration^[^1–5,7^]^. Our interpretation is that because the patient was unable to tolerate even liquids due to complete duodenal obstruction, and because the sigmoid mass did not cause complete colonic obstruction, the typical clinical manifestations of large-bowel obstruction, such as abdominal distension and constipation, did not become apparent.

Retroperitoneal involvement is an uncommon presentation. In very rare cases, malignant retroperitoneal infiltration can mimic benign conditions such as RPF^[^4,7,8,10,11^]^. This can further complicate the diagnostic process, especially in our case, with a known contrast-media allergy, no prior GI symptoms, and no history of colorectal screening.

### Review of RPF case reports

A few case reports in the literature have described RPF as increasingly associated with underlying malignancies. Reported cases include a retrospective analysis of 111 patients with RPF that revealed a strong correlation between RPF and malignancies (overall standardized incidence ratio of 2.2 compared with the general population, and a higher risk of malignancy within the first year after diagnosis of RPF)^[^11^]^. Another case describes a 56-year-old woman with gastric adenocarcinoma who presented with bilateral hydronephrosis and acute kidney injury secondary to RPF, and while initial diagnostic approaches revealed a primary gastric lesion, biopsy confirmed malignant infiltration; this case emphasizes the need to consider occult malignancy in patients with bilateral ureteral obstruction and RPF^[^12^]^. Another case reports a 25-year-old male with post-surgical RPF secondary to a sigmoid malignancy who developed chronic renal failure despite receiving treatment for the malignancy^[^7^]^.

However, most of the reported cases focus on patients with known primary malignancies or that present with isolated malignant ureteral obstruction secondary to colorectal cancer^[^12,13^]^, or with isolated partial GOO following recurrent colon adenocarcinoma^[^14^]^. What makes our case unique is the simultaneous presentation of both dialysis-dependent obstructive uropathy and GOO, especially in a patient without prior signs or symptoms of GI cancer.

### Pathophysiological explanation

Colorectal cancer can metastasize to different regions in the body through the lymphatic pathway or direct peritoneal seeding^[^4,15,16^]^. This tumor infiltration can trigger a significant desmoplastic reaction, which in turn leads to the formation of fibrotic tissues. These fibrotic tissues may mimic idiopathic RPF in diagnostic modalities^[^16,17^]^. Depending on the location of the tissue formed, the compressive effect may cause multiorgan obstruction of the adjacent structures. In our patient, the retroperitoneal lesion was confirmed histologically as adenocarcinoma and was found intraoperatively to encase nearby structures. For instance, ureteral obstruction may lead to bilateral hydronephrosis progressing to renal failure. According to the severity of the obstruction, hemodialysis may be required to prevent the accumulation of toxins in the bloodstream^[^7,12,18^]^.

Furthermore, RPF can compress the second and third portions of the duodenum, leading to mechanical GOO and promoting nausea and severe vomiting. The development of both GI and renal obstructions reveals an aggressive infiltrative pattern rather than simple metastatic nodules ^[^7,10,19^]^.

### Diagnostic challenges and management

The differential diagnosis of RPF includes idiopathic causes (particularly IgG4-related disease), secondary inflammatory or infectious causes (such as tuberculosis), prior surgery or radiation, drug-induced fibrosis, and malignant retroperitoneal infiltration^[^20–22^]^. In our patient, the absence of clinical features suggestive of systemic IgG4-related disease or chronic infection, the progressive obstructive course, failure of ureteral stenting, suspicious pulmonary nodules, and subsequent histopathological confirmation supported a diagnosis of metastatic adenocarcinoma rather than a benign cause.

This case presents significant diagnostic and therapeutic challenges because of its atypical presentation with misleading imaging findings. Because of an allergy to contrast media in our patient, we were unable to perform contrast-enhanced CT, which limited the accurate detection of the retroperitoneal process. Both noncontrast CT and MRI suggested a benign condition, which delayed the correct diagnosis of the underlying malignancy.

Presenting initially with bilateral hydronephrosis and AKI symptoms raised suspicion of a urological etiology. However, when the patient does not significantly improve after a double-J stent insertion, this suggests ureteral encasement^[^7,12,23^]^. In addition, confirmation of GOO after re-evaluation of imaging in our patient supported the presence of a broader infiltrative process.

Such a case warranted a multispecialty approach. As double-J stenting failed to achieve the desired outcomes, percutaneous nephrostomy was performed^[^7,23^]^. Due to the severity of the GOO, surgical intervention was performed to relieve the obstruction and explore the abdomen. A peritoneal biopsy was obtained, and the primary sigmoid tumor was identified.

This emphasizes the importance of early detection and timely intervention in such complicated cases. This case highlights the need for reconsideration of the diagnosis, especially when standard management techniques fail. Although our patient lacked typical large-bowel obstruction symptoms, this case also highlights the importance of considering colonoscopic evaluation when the etiology of RPF or infiltrative retroperitoneal disease remains uncertain. Colonoscopy may identify an otherwise occult colorectal primary lesion and facilitate earlier diagnosis, staging, and treatment planning^[^24,25^]^.

### Take-home messages and limitations

First, RPF should be considered a diagnosis of exclusion, especially in patients with a rapidly deteriorating condition. Second, failure of standard approaches such as double-J stenting warrants further investigation for malignant encasement. Third, multi-level obstruction raises suspicion of an aggressive, infiltrative malignant process.

The limitations in our case included the inability to perform contrast-enhanced imaging, which in turn limited the ability to assess the boundaries of tumor infiltration in detail before surgery.

## Conclusion

Occult sigmoid adenocarcinoma can present with extraluminal manifestations in the absence of typical GI symptoms. In patients with unexplained RPF, particularly those with progressive obstruction or poor response to treatment, colonoscopy should be considered to exclude an occult colorectal malignancy, even in the absence of typical GI symptoms. Additionally, retroperitoneal metastasis can mimic RPF on imaging, especially when contrast media are contraindicated. Finally, early diagnosis and prompt interventions require multidisciplinary collaboration to achieve desired outcomes and improve patient survival.

## Data Availability

The data supporting the findings of this case report are available from the corresponding author upon request. The data are not publicly available due to privacy and ethical restrictions.
